# Transient dynamics of a single molecular transistor in the presence of local electron–phonon and electron–electron interactions and quantum dissipation

**DOI:** 10.1038/s41598-022-13032-4

**Published:** 2022-06-08

**Authors:** Manasa Kalla, Narasimha Raju Chebrolu, Ashok Chatterjee

**Affiliations:** 1 School of Physics, University of Hyderabad, Hyderabad, 500046 India; 2 Department of Physics, Central University of Karnataka, Kalaburagi, 585367 India

**Keywords:** Condensed-matter physics, Electronics, photonics and device physics, Quantum physics

## Abstract

We consider a single molecular transistor in which a quantum dot with local electron–electron and electron–phonon interactions is coupled to two metallic leads, one of which acts like a source and the other like a drain. The system is modeled by the Anderson-Holstein (AH) model. The quantum dot is mounted on a substrate that acts as a heat bath. Its phonons interact with the quantum dot phonons by the Caldeira–Leggett interaction giving rise to dissipation in the dynamics of the quantum dot system. A simple canonical transformation exactly treats the interaction of the quantum dot phonons with the substrate phonons. The electron–phonon interaction of the quantum dot is eliminated by the celebrated Lang-Firsov transformation. The time-dependent current is finally calculated by the Keldysh Green function technique with various types of bias. The transient-time phase diagram is analysed as a function of the system parameters to explore regions that can be used for fast switching in devices like nanomolecular switches.

## Introduction

With the advances in fabrication techniques in nanotechnology, together with the availability of ultrafast experiments to analyze the out-of-equilibrium quantum dynamics of many body interacting systems, research in molecular electronics has gathered unprecedented momentum in recent years. Indeed, ultrafast experiments have been employed to study the charge transport in a three-terminal device like a single molecular transistor (SMT)^1–7^. Recently, Cocker et al.^8^ have studied the charge transport dynamics through a singly-occupied localized orbital, like highest occupied molecular orbital (HOMO) in pentacene molecule using the terahertz-scanning tunneling microscope (THz-STM) technique. Many experimental groups have fabricated molecular devices using the C_60_ molecule^9–11^ and found that the current is controlled effectively by tuning the gate voltage. Also, many researchers have reported the non-equilibrium effects of electron–phonon (*e–p*) interaction during the charge tunneling in molecular devices like phonon-assisted tunneling transport^12–22^, hysteresis-induced bistability^23^, local heating^24^, molecular switching^25,26^, and negative differential conductance^27–29^. Chen et al.^30^ have studied the polaronic effects on the current density using the Keldysh formalism. They have reported that the current density decreases with increasing the *e–p* interaction and also the formation of phonon side peaks in the spectral density. Later, Raju and Chatterjee^31^ have studied the effects of quantum dissipation due to substrate in an SMT device with electron–electron (*e–e*) and *e–p* interactions and reported that the dissipation enhances the current density. In recent work, we have investigated the magneto-transport properties^32^ of an SMT system and explained that it can be used as a spin-filtering device at zero temperature. We have also shown the effect of *e–p* interaction on the transport properties at finite temperature in^33^.

Of late, the transient dynamics of mesoscopic systems has attracted considerable attention. In this context, dissipative driven quantum rings, dynamical Franz-Keldysh effect, molecular electronics have been extensively studied. The theoretical investigation of transient dynamics in molecular electronic devices with strong *e–p* interaction is a challenging task. It is essential from the point of view of the application of these devices for quantum computing. Jauho et al*.*^34^ have analyzed the transient dynamics through a non-interacting quantum dot (QD) response to harmonic and sharp square-shaped voltage pulses by using the Keldysh Green function method. Schmidt et al*.*^35^ have investigated the time-dependent transport through QD with weak Coulomb interaction using the Anderson model within the framework of a mean-field approximation and used the Monte Carlo technique to calculate the current density. Many researchers^36–38^ have investigated the transient dynamics of QD modeled by the impurity Anderson model in the Kondo regime by employing different approaches like the time-dependent noncrossing approximation method^39,40^, time-dependent density matrix renormalization group technique^41^, functional renormalization group approach^42^. In reference^43^, they studied the transient dynamics of a single molecular junctions modeled with the Anderson-Holstein model using the auxiliary-mode expansion method . In the present work, we study the transient dynamics of an SMT device with *e–e* and *e–p* interactions and quantum dissipation by employing Keldysh formalism. Here we explore the transient dynamics of the SMT device with two different time modulations, namely, the harmonic pulse modulation and the upward pulse modulation.

## Model

**Figure 1:**
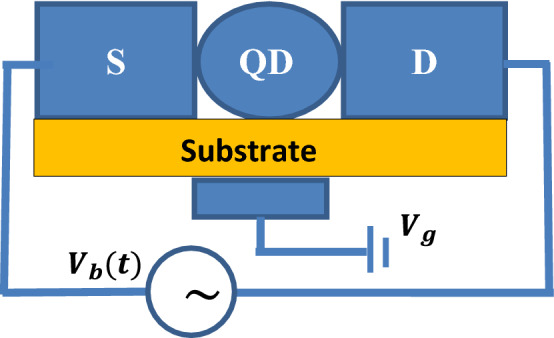
Schematic diagram of an SMT system.

Figure 1 represents a block diagram of an SMT device with a molecule or QD is coupled to two metallic leads, a source ($S$) on the left and a drain ($D$) on the right. The entire structure is mounted on an insulator (substrate), which acts like a phonon bath. The electron energy levels in the QD system can be tunable by changing the gate voltage ${V}_{g}$. The system is driven by the bias voltage ${V}_{b}$ which is considered to be time-dependent.

To model the system under consideration, we consider the extended AH model Hamiltonian by incorporating the Caldeira-Leggett^44^ term to take care of the dissipation. The Hamiltonian $H$ can be written as:

1 $$H= {H}_{l}+{H}_{c}+{H}_{t}+{H}_{B} .$$

Here ${H}_{l}$ is the Hamiltonian describing the leads and is given by

2 $${H}_{l}=\sum_{\begin{array}{c}k,\sigma \\ \alpha \epsilon S,D\end{array}}{\varepsilon }_{\alpha k\sigma }\left(t\right) {n}_{\alpha k\sigma } ,$$

where ${n}_{\alpha k\sigma }(={c}_{\alpha k\sigma }^{\dagger}{c}_{\alpha k\sigma })$ gives the number of the conduction electrons in the lead $\alpha$ with momentum $k$, with time-dependent energy**,** ${\varepsilon }_{\alpha k\sigma }(t)={\varepsilon }_{\alpha k\sigma }^{0}+{\Delta }_{\alpha }\left(t\right)$, ${\Delta }_{\alpha }\left(t\right)$ being the time-dependent bias which is equal to ${V}_{b}\left(t\right)$ and ${c}_{\alpha k\sigma }({c}_{\alpha k\sigma }^{\dagger})$ is the corresponding electron annihilation (creation) operator. The second term in the Hamiltonian ${H}_{c}$ models the central region or the QD and is given by

3 $${H}_{c}=\sum_{\sigma =\uparrow ,\downarrow }\left({\varepsilon }_{d}\left(t\right)-{eV}_{g}\right) {n}_{d\sigma }+U{n}_{d,\sigma }{n}_{d,-\sigma }+\hslash {\omega }_{0}{b}^{\dagger}b+ \lambda \hslash {\omega }_{0}\left({b}^{\dagger}+b\right)\sum_{\sigma }{n}_{d\sigma } ,$$

where ${n}_{d\sigma }\left(={c}_{d\sigma }^{\dagger}{c}_{d\sigma }\right)$ describes the number operator for the central dot electrons with time-dependent energy ${\varepsilon }_{d}$,${c}_{d\sigma } ({c}_{d\sigma }^{\dagger})$ represents the dot electron annihilation (creation) operator, $U$ is the onsite *e–e* interaction, ${b}^{\dagger}(b)$ is the creation (destruction) operator for a QD phonon with frequency ${\omega }_{0}$ and $\lambda$ is the measure of *e–p* interaction strength. The third term in Hamiltonian, ${H}_{t}$ represents the tunneling coupling between the QD and leads with coupling strength ${V}_{k}\left(t\right)$, can be written as

4 $${H}_{t}=\sum_{\begin{array}{c}k,\sigma \\ \alpha \epsilon S,D\end{array}}\left({V}_{k}\left(t\right){c}_{\alpha k\sigma }^{\dagger}{c}_{d\sigma }+h.c\right)$$

Finally the ${H}_{B}$ term describes the substrate phonons and their interaction with the phonon of the QD, is given by

5 $${H}_{B}=\sum_{j=1}^{N}\left[\frac{{p}_{j}^{2}}{2{m}_{j}}+\frac{1}{2}{m}_{j}{\omega }_{j}^{2}{x}_{j}^{2 }\right]+\sum_{j=1}^{N}{\beta }_{j}{x}_{j}{x}_{0}$$

where ${x}_{j}$ and ${x}_{0}$ denotes the generalized coordinate of the substrate and dot oscillator, ${\omega }_{j}$ gives the frequency of the $j$th substrate oscillator and ${\beta }_{j}$ represents the linear coupling strength between the $j$th oscillator of the substrate and dot oscillator.

## Formulation

### Elimination of the interaction with bath phonons

The linear interaction between the dot and bath oscillators can be decoupled by performing the following unitary transformation

6 $${\tilde{x }}_{j}= {x}_{j}+\frac{{\beta }_{j}{x}_{0}}{{m}_{j}{w}_{j}^{2}} , {\tilde{p }}_{j}=-i\hslash \frac{\partial }{\partial {\tilde{x }}_{j}} .$$

As a result, the local phonon frequency of QD ${\omega }_{0}$ gets renormalize to ${\stackrel{\sim }{\omega }}_{0}$, is given by ${\stackrel{\sim }{\omega }}_{0}={\left({\omega }_{0}^{2}-\Delta {\omega }^{2}\right)}^{1/2}$, where $\Delta {\omega }^{2}\left(=\sum_{j=1}^{N}{\beta }_{j}^{2}/\left({m}_{0}{m}_{j }{{\omega }_{j}}^{2}\right)\right)$ is the change in ${\omega }_{0}^{2}$ due to the interaction with the bath phonons. The decoupled Hamiltonian is given by

7 $${H}_{vib}+{H}_{B}=\left(\frac{{p}_{0}^{2}}{2{m}_{0}}+\frac{1}{2} {m}_{0}{{\stackrel{\sim }{\omega }}_{0}}^{2}{x}_{0}^{2}\right) +\sum_{j=1}^{N}\left(\frac{{\tilde{p }}_{j}^{2}}{2{m}_{j}}+\frac{1}{2} {m}_{j}{{\omega }_{j}}^{2}{{\tilde{x }}_{j}}^{2}\right).$$

Here onwards we will discard the bath Hamiltonian because it merely adds constant energy to the system. We considered the spectral function $J\left(\omega \right)$ to describe the dynamics of the substrate phonons which is given by $J\left(\omega \right)=\sum_{j=1}^{N} \frac{{\beta }_{j}^{2}}{2{m}_{j}{\omega }_{j}} \delta \left(\omega -{\omega }_{j}\right).$ For all frequencies $\omega$, the spectral function obeys the relation in the strict Ohmic case: $J\left(\omega \right)=2{m}_{0}\gamma \omega$, where the ohmic damping coefficient: $\upgamma =\frac{1}{2{m}_{0}}\sum_{j=1}^{N}\left({\beta }_{j}^{2}/2{m}_{j}{\omega }_{j}^{2}\right)\delta \left(\omega -{\omega }_{j}\right)$. The pure Ohmic spectral density calculated above, however, is not very practical because it diverges in the limit of $\omega \to \infty$. In order to salvage the situation, we have considered the Lorentz-Drude form^45^ for $J\left(\omega \right)$ with the cut of frequency ${\omega }_{c}$,

8 $$J\left(\omega \right)=\frac{2{m}_{0}\gamma \omega }{\left[1+{\left(\frac{\omega }{{\omega }_{c}} \right)}^{2}\right]} ,$$

in the limit $\omega \to \infty$, $J\left(\omega \right)$ approaches the value zero and in the small-$\omega$ limit, we can obtain the pure Ohmic spectral density. The final expression for ${\stackrel{\sim }{\omega }}_{0}$ become, ${\stackrel{\sim }{\omega }}_{0}={\left({\omega }_{0}^{2}-\Delta {\omega }^{2}\right)}^{1/2} ,$ where $\Delta {\omega }^{2}=2\pi {\gamma \omega }_{c}.$

The total transformed Hamiltonian reads as,

9 $$\overline{H }=\sum_{\begin{array}{c}k,\sigma \\ \alpha \epsilon S,D\end{array}}{\varepsilon }_{\alpha k\sigma }\left(t\right){n}_{k\alpha }+\sum_{\sigma =\uparrow ,\downarrow }\left({\varepsilon }_{d}\left(t\right)-{eV}_{g}\right) {n}_{d\sigma }+U{n}_{d,\sigma }{n}_{d,-\sigma }+\mathrm{\hslash }{\stackrel{\sim }{\omega }}_{0}{b}^{\dagger}b+ \lambda \mathrm{ \hslash }{\stackrel{\sim }{\omega }}_{0}\left({b}^{\dagger}+b\right)\sum_{\sigma }{n}_{d\sigma }+ \sum_{\begin{array}{c}k,\sigma \\ \alpha \epsilon S,D\end{array}}\left({V}_{k}\left(t\right){c}_{\alpha k\sigma }^{\dagger}{c}_{d\sigma }+h.c\right) .$$

### Elimination of *e–p* coupling: Lang-Firsov Transformation (LFT)

To decouple the *e–p* interaction, we apply on the Hamiltonian $\overline{H }$, the celebrated LFT^46^ is given by the unitary operator: $U={e}^{S},$ where the anti-Hermitian generator $S$ is given by:

10 $$S=\lambda \left({b}^{\dagger}-b\right)\sum_{\sigma }{n}_{d\sigma }.$$

The transformed Hamiltonian becomes

11 $$\tilde{H }={e}^{S}\overline{H}{e }^{-S}=\sum_{\begin{array}{c}k\sigma \\ \alpha \epsilon S,D\end{array}}{\varepsilon }_{\alpha k\sigma }\left(t\right){n}_{k\sigma }+\sum_{\sigma }\left({\stackrel{\sim }{\varepsilon }}_{d}\left(t\right)-{eV}_{g}\right){n}_{d\sigma }+ \tilde{U }{n}_{d,\sigma }{n}_{d,-\sigma }+\mathrm{\hslash }{\stackrel{\sim }{\omega }}_{0}{b}^{\dagger}b+\sum_{\begin{array}{c}k,\sigma \\ \alpha \epsilon S,D\end{array}}({\tilde{V }}_{k}\left(t\right){c}_{\alpha k\sigma }^{\dagger}{c}_{d\sigma }+h.c)$$

where

12 $${\stackrel{\sim }{\varepsilon }}_{d\sigma }\left(t\right)={\varepsilon }_{d}\left(t\right)-e{V}_{g}-{\lambda }^{2}\hslash {\stackrel{\sim }{\omega }}_{0} , \tilde{U }=U-2{\lambda }^{2}\hslash {\stackrel{\sim }{\omega }}_{0} , {\tilde{V }}_{k}\left(t\right)={V}_{k}\left(t\right){e}^{\lambda \left({b}^{\dagger}-b\right)} ,$$

### Tunneling current: The Keldysh formalism

The time-dependent tunneling current^34^ of the SMT system can be written as

13 $${J}_{\alpha }\left(t\right)=- \frac{e}{\hslash } {\Gamma }_{\alpha }\left[N\left(t\right)+\frac{1}{\pi } {\int }_{-\infty }^{\infty }d\varepsilon {f}_{\alpha }\left(\varepsilon \right) \mathrm{Im}\left({A}_{\alpha }\left(\varepsilon ,t\right)\right)\right] ,$$

where

14 $${f}_{\alpha }\left(\varepsilon \right)=\frac{1}{\left[{e}^{({\mu }_{\alpha }-\varepsilon )/{k}_{B}T}+1\right] },$$

is the Fermi distribution of the lead $\alpha$ and ${\mu }_{\alpha }$ is the corresponding chemical potential which is related to ${V}_{b}$ as:

${\mu }_{\alpha }=e{V}_{b} {\delta }_{\alpha S}$, here ${\delta }_{\alpha S}$ is the Kronecker delta function and ${\Gamma }_{\alpha }$ is given by

15 $${\Gamma }_{\alpha }= \sum_{\begin{array}{c}\alpha \epsilon S,D\\ n\end{array}}{\Gamma }_{\alpha }\left(\varepsilon \right) \frac{{\lambda }^{2n}{e}^{-{\lambda }^{2}}}{n!} ,$$

with

16 $${\Gamma }_{\alpha }\left(\varepsilon \right)=2\pi {\rho }_{s,D}\left(\varepsilon \right) {\overline{\tilde{V }} }_{k}{V}_{k}^{*}.$$

Within the local polaron approximation we can replace, ${\overline{\tilde{V }} }_{k}=\langle {\tilde{V }}_{k}\left(t\right)\rangle ={V}_{k}\left(t\right) {e}^{-{\lambda }^{2}({N}_{ph}+1/2)}$, ${N}_{ph}=1/[{e}^{\beta \mathrm{\hslash }{\stackrel{\sim }{\omega }}_{0}}-1]$ represents the phonon population, and at zero temperature ${\overline{\tilde{V }} }_{k}={V}_{k}\left(t\right) {e}^{-{\lambda }^{2}/2}.$ Here, our numerical computation is done at zero temperature which also evident that we have considered that the local phonons are always in thermal equilibrium with the bath phonons. Where ${\rho }_{S\left(D\right)}$ represents the constant density of states in the source (drain). Within the wide-band approximation, ${\rho }_{s,D}\left(\varepsilon \right)$ are independent of energy, and ${N}_{d,\sigma }\left(t\right)$ is the occupancy of the energy levels in QD, which is given by

17 $${N}_{d,\sigma }\left(t\right)=\sum_{\alpha ,n}{\Gamma }_{\alpha }\underset{-\infty }{\overset{\infty }{\int }}\frac{d\varepsilon }{2\pi }{f}_{\alpha }\left(\varepsilon \right) {\left|{A}_{\alpha ,\sigma }\left(\varepsilon +n\hslash {\stackrel{\sim }{\omega }}_{0},t\right)\right|}^{2} .$$

In ${N}_{d,\sigma }\left(t\right),$ ${A}_{\alpha }\left(\varepsilon ,t\right)$ is the spectral function of the dot, which is calculated by using the Keldysh Green function method as

18 $${A}_{\alpha ,\sigma }\left(\varepsilon ,t\right)= {\int }_{-\infty }^{t}d{t}_{1}{G}_{d,\sigma }^{r}\left(t,{t}_{1}\right) exp\left[i \varepsilon \left(t-{t}_{1}\right)-i{\int }_{t}^{{t}_{1}}d{t}_{2}{\Delta }_{\alpha }\left({t}_{2}\right)\right] ,$$

where ${G}_{d,\sigma }^{r}\left(t,{t}_{1}\right)$ is the retarded Green function given by

19 $${G}_{d,\sigma }^{r}\left(t,{t}_{1}\right)=-i\theta \left(t-{t}_{1}\right) \langle \left\{{c}_{d\sigma }\left(t\right),{c}_{d\sigma }^{\dagger}\left({t}_{1}\right)\right\}\rangle .$$

Using the equation of motion method, we get the expression for ${G}^{r}\left(t,{t}_{1}\right)$ as

20 $${G}_{d,\uparrow (\downarrow )}^{r}\left(t,{t}_{1}\right)=-i\theta \left(t-{t}^{^{\prime}}\right) exp\left[-i\int \left({\stackrel{\sim }{\varepsilon }}_{d}-\tilde{U }\langle {N}_{d\downarrow (\uparrow )}\left(t\right)\rangle +i\frac{{\Gamma }_{\alpha }}{2}\right)\left(t-{t}_{1}\right) d{t}_{1}\right] .$$

To get the above expression we have treated the *e–e* interaction in the mean field level, i.e. $\langle \left\{{c}_{d\uparrow }^{\dagger}\left(t\right){c}_{d\uparrow }\left(t\right){n}_{d\downarrow }\left(t\right), {c}_{d\uparrow }^{\dagger}\left({t}_{1}\right)\right\}\rangle \cong \langle {n}_{d\downarrow }\left(t\right)\rangle \langle \left\{{c}_{d\uparrow }^{\dagger}\left(t\right){c}_{d\uparrow }\left(t\right), {c}_{d\uparrow }^{\dagger}\left({t}_{1}\right)\right\}\rangle$. Here we investigate the time-dependent current through the system for two time modulations.

**(i) Harmonic pulse time modulation**

In the case of harmonic pulse modulation, ${\Delta }_{\alpha }\left(t\right)={\Delta }_{\alpha }\mathrm{cos}\omega t$ and substituting in Eq. (18) and after some algebraic manipulation, we obtain the imaginary part of corresponding spectral function as,

21 $$Img\left({A}_{\alpha ,\uparrow (\downarrow )}\left(\varepsilon ,t\right)\right)= -\sum_{k=-\infty }^{\infty }{J}_{k}^{2}\left(\frac{{\Delta }_{\alpha }}{\omega }\right)\frac{\left(\frac{{\Gamma }_{\alpha }}{2}\right)}{\left[{\left(\varepsilon -{\stackrel{\sim }{\varepsilon }}_{d}-n\hslash {\stackrel{\sim }{\omega }}_{0}-\tilde{U }\langle {N}_{d,\downarrow (\uparrow )}\left(t\right)\rangle -k\omega \right)}^{2}+{\left(\frac{{\Gamma }_{\alpha }}{2}\right)}^{2}\right]} ,$$

where ${J}_{k}\left({\Delta }_{\alpha }/\omega \right)$ is the Bessel function of the first kind and $\omega =2\pi /T$,$T=(\pi \hslash /\Gamma )$ being the time period of the harmonic bias voltage.

**(ii) Upward pulse time modulation**

In this case, we consider ${\Delta }_{\alpha }\left(t\right)={\Delta }_{\alpha } u\left(t\right)$, where $u\left(t\right)$ is the Heaviside function and the corresponding spectral function is calculated as

22 $${A}_{\alpha }\left(\varepsilon ,t\right)=\frac{\left(\varepsilon -{\stackrel{\sim }{\varepsilon }}_{d}+\tilde{U }\langle {N}_{d,\downarrow }\left(t\right)\rangle +i\frac{{\Gamma }_{\alpha }}{2}\right)+{\Delta }_{\alpha }exp\left[i\left(\varepsilon +{\stackrel{\sim }{\varepsilon }}_{d}+\tilde{U }\langle {N}_{d,\downarrow }\left(t\right)\rangle +{\Delta }_{\alpha }+i\frac{{\Gamma }_{\alpha }}{2}\right)t\right]}{\left[{\left(\varepsilon +\tilde{U }\langle {N}_{d,\downarrow }\left(t\right)\rangle +i\frac{{\Gamma }_{\alpha }}{2}\right)}^{2}-{\stackrel{\sim }{\varepsilon }}_{d}^{2}\right]} .$$

Substituting the imaginary part of the above function in Eq. (13) finally, yields the time-dependent current density.

## Results and discussions

In the present work, we consider a single energy level in QD with energy ${\varepsilon }_{d}=0.5,$ and symmetric coupling $({\Gamma }_{S}= {\Gamma }_{D}=\Gamma /2$) of the dot level with the source and drain. Here, we measure all the energy quantities in terms of dot phonon energy $\hslash {\omega }_{0}$, time in units of $\hslash /\Gamma$ and in rest of the paper we set $\hslash =1$. Here we have studied the normalized current $J/{J}_{0}=({J}_{S}-{J}_{D})$ with ${J}_{0}=e\Gamma /2\hslash$ for two different input pulses*.* Here we have taken $e{V}_{g}=0.3, {\gamma =0.03, \omega }_{c}=5,\Gamma =0.2, {eV}_{b}=0.5, { k}_{B}T=0, U=3$, ${\Delta }_{D}=0$ and ${\Delta }_{S}=5$. We first present our results for the Harmonic pulse and then we will discuss the results for the upward pulse.

### Harmonic Pulse modulation

**Figure 2:**
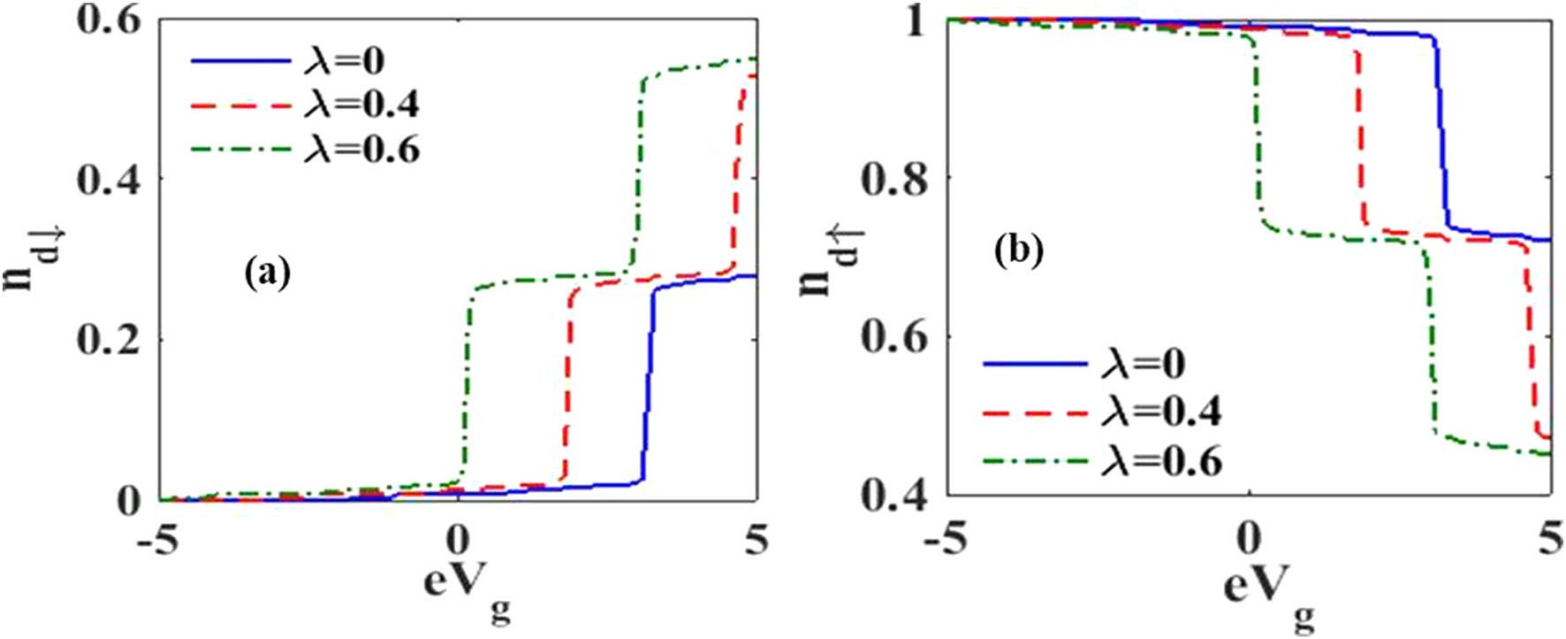
Dot occupancy versus gate voltage for different values of *e–p* interaction strengths.

Figure 2 represents the behavior of occupancy of the dot as a function of gate voltage for various *e–p* coupling strengths at $t=10$. We have calculated the values of ${N}_{d\uparrow }$ and ${N}_{d\downarrow }$ self-consistently subjected to the condition that ${N}_{d\uparrow }+{N}_{d\downarrow }=1.$ The occupancy clearly displays the staircase structure due to phonon side bands. The up-spin occupancy ${N}_{d\uparrow }$ increases with increasing gate voltage while ${N}_{d\downarrow }$ shows a decreasing behavior.

**Figure 3:**
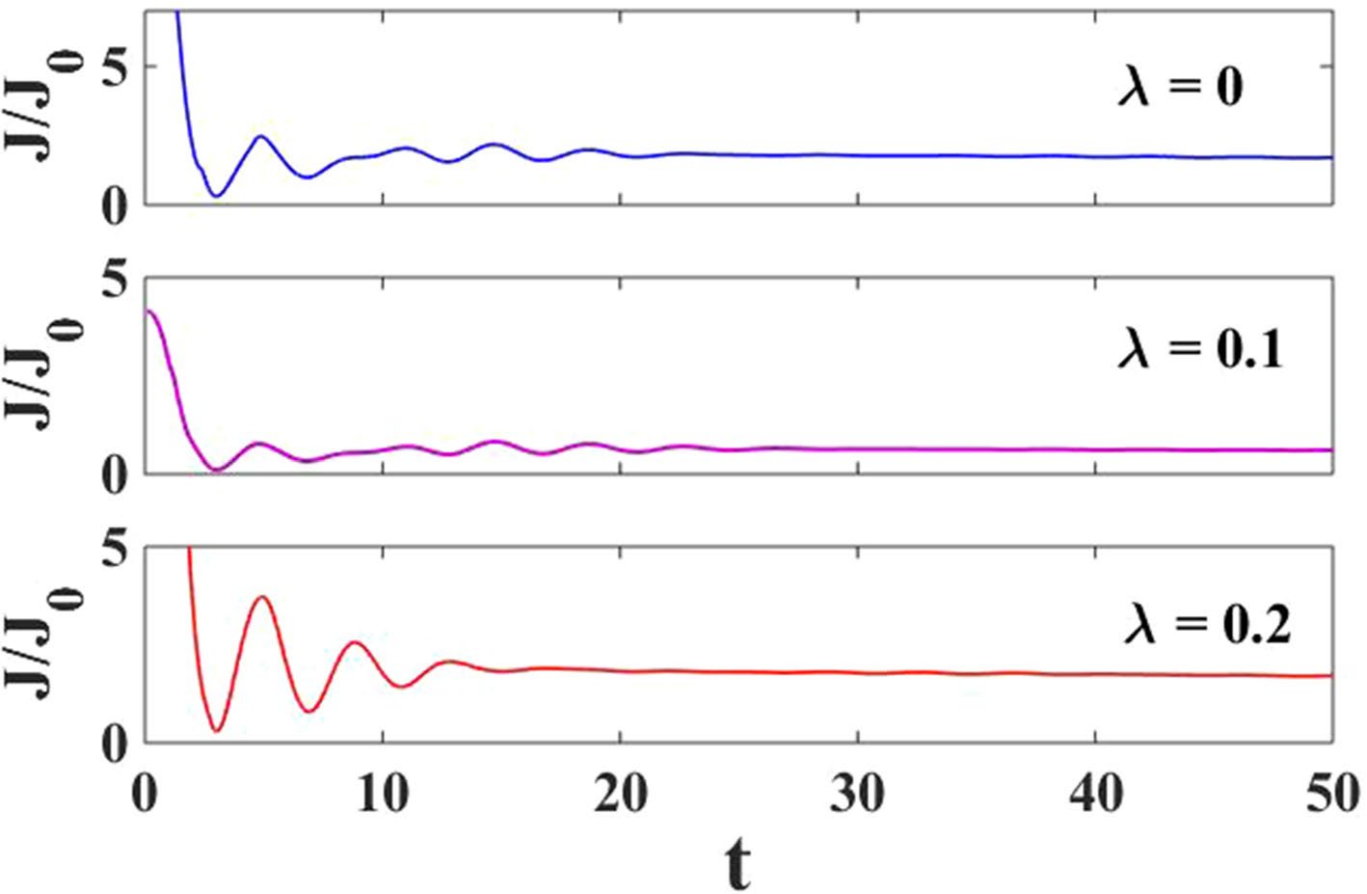
$J/{J}_{0}$ versus $t$ for various values of $\lambda$ at ${eV}_{g}=0.6$.

In Fig. 3, we have plotted the normalized current density versus time for different $\lambda$ values with ${J}_{0}=e\Gamma /2$. Here, one can observe that the oscillations in the normalized current decrease with increasing time and reach a steady state at a particular time called transient time. We can observe that the transient time decreases with increasing *e–p* interaction strength and it can be admitted to variations in the hopping parameters. The reason can be explained as when an electron travels from the source to the dot, and it interacts with the phonons as a result, it forms a polaron. The polaronic effects can be clearly observed from Eqs. (12) and (15). The current density approaches the steady-state faster as the *e–p* interaction strength increases as a result the transient time decreases.

**Figure 4:**
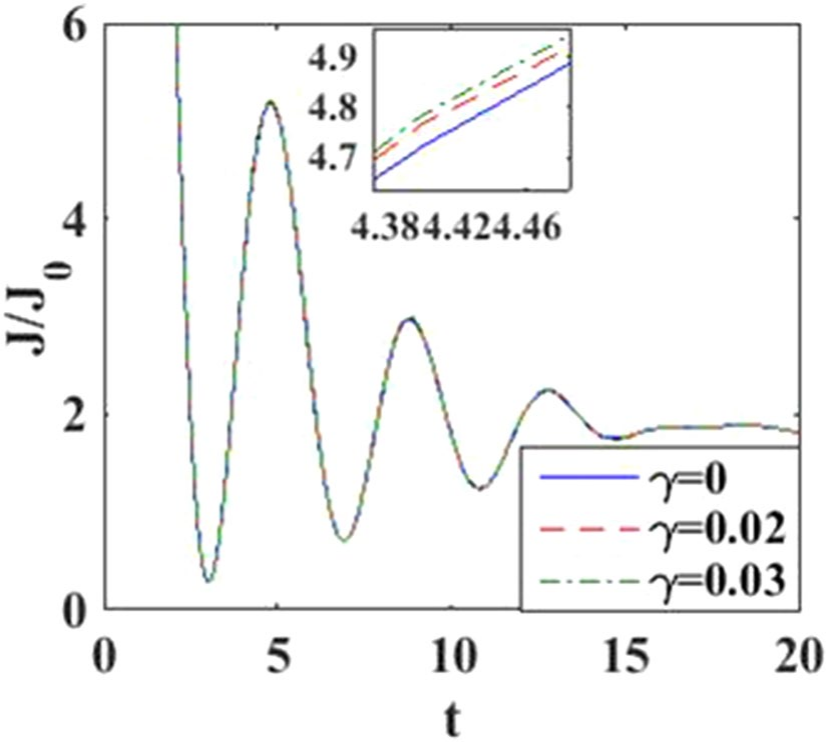
$J/{J}_{0}$ versus $t$ for various values of damping rate at $\lambda =0.6$.

**Figure 5:**
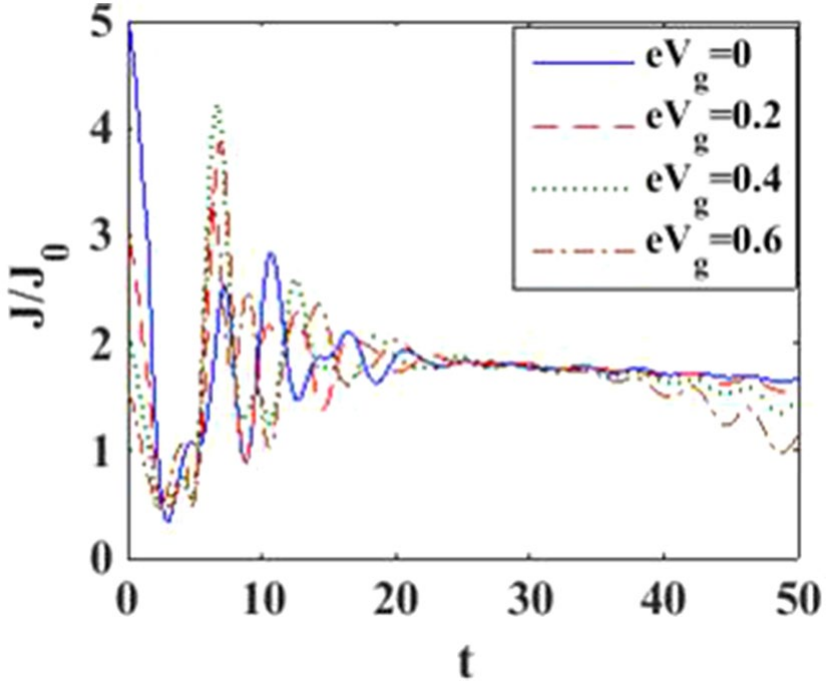
$J/{J}_{0}$ versus $t$ for various values of ${eV}_{g}$ at $\lambda =0.6$.

**Figure 6:**
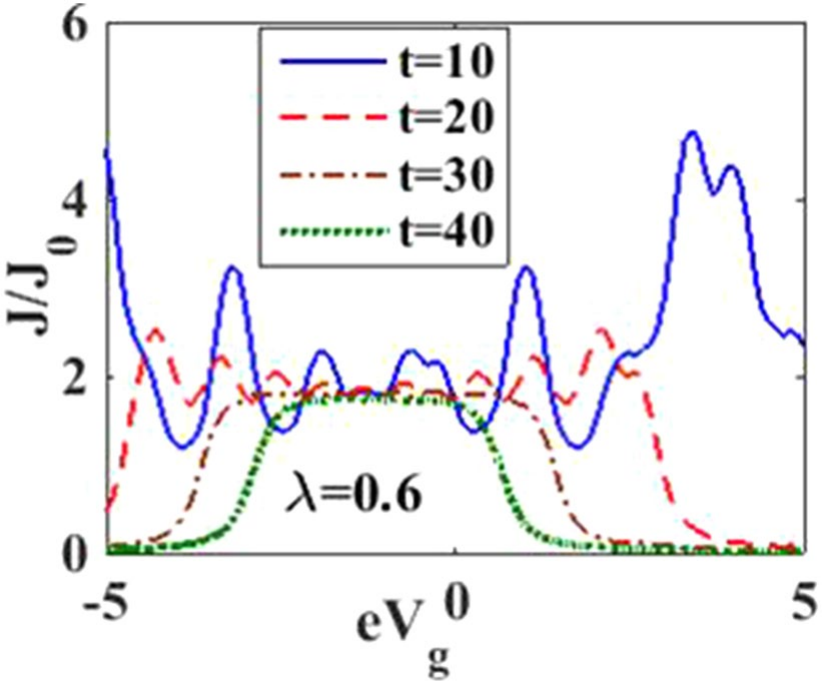
$J/{J}_{0}$ versus ${eV}_{g}$ for various values of time at ${eV}_{b}=0.5$.

**Figure 7:**
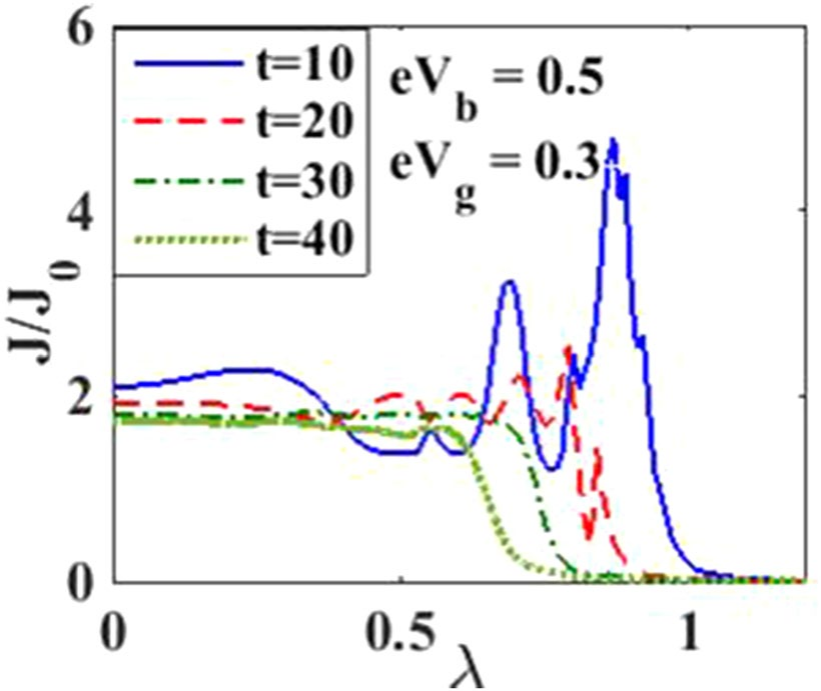
$J/{J}_{0}$ versus $\lambda$ for various values of time.

**Figure 8:**
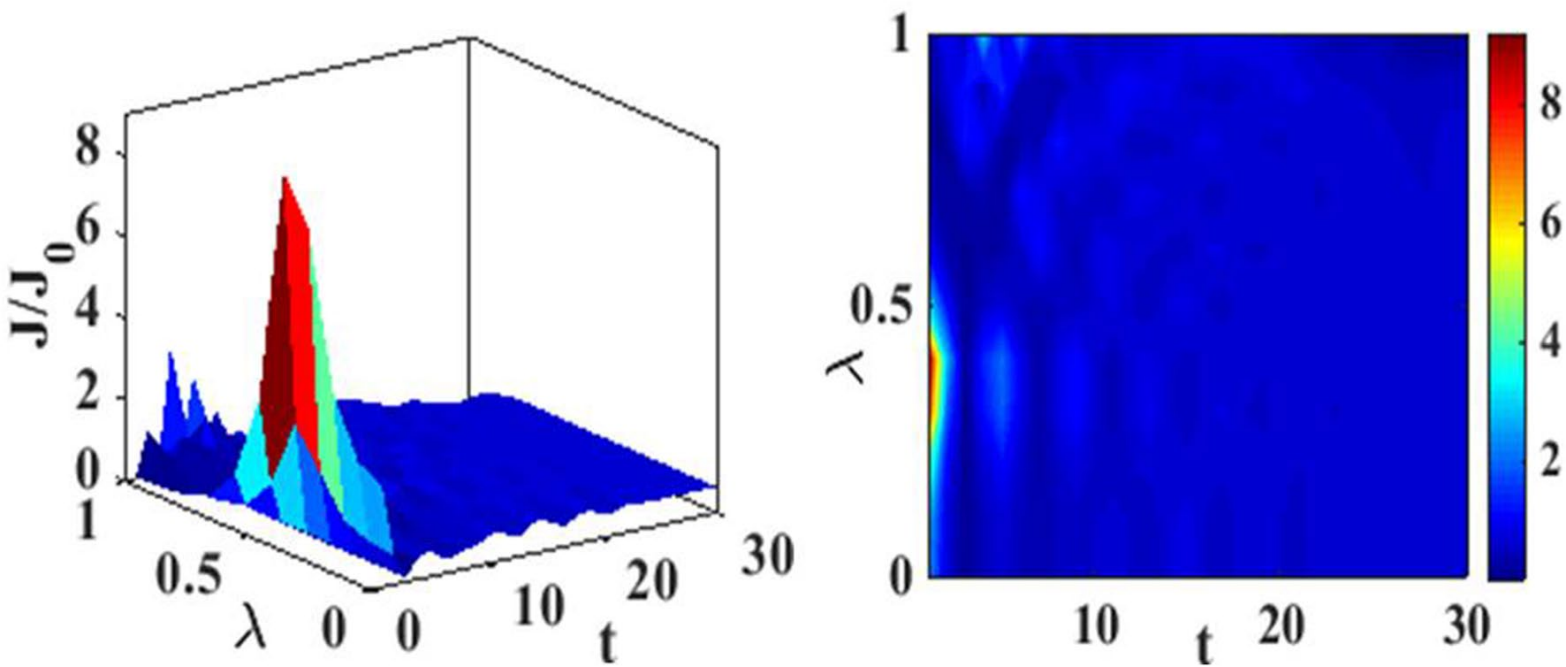
Current density as a function of $t$ and $\lambda$. Three-dimensional plot (left) and contour map (right)$.$

**Figure 9:**
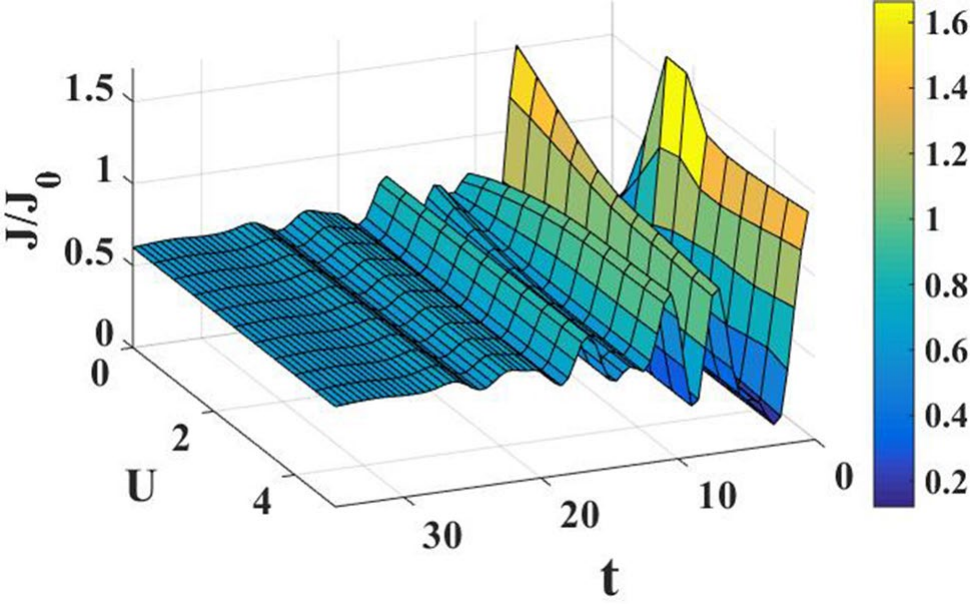
Contour map of the current density as a function of $t$ and $U$ at $\lambda =0.4, e{V}_{g}=0.6$.

In Fig. 4, we have plotted the current density as a function of time for different values of the damping rate. One can observe from the inset that the damping rate slightly increases the current density, which is expected behavior. We have presented the current density versus time result for different gate voltages in Fig. 5 to see the dependence of transient time on the gate voltage. But it is not very clear from the figure how the transient time changes with gate voltage, though it appears to decrease with increasing gate voltage up to a certain time. To have more clarity, we plot the current density versus gate voltage for different time values in Fig. 6. It is evident that as time starts, the current density fluctuates much more with increasing gate voltage, while as time advances sufficiently, the fluctuations subside. In Fig. 7, we show the current density versus $\lambda$ result for different time values. One would expect that the normalized current density would decrease with increasing $\lambda$ due to polaronic effects. However, many fluctuations seem to occur at a small time and one has to actually wait for some time to get the stable value of the current density. In Fig. 8, we show the three-dimensional plot and the contour map of the current density as a function of $t$ and $\lambda$. It is clear from the figure that $\lambda$ reduces the transient time, same as observed in Fig. 3. Figure 9 represents the contour map of the current density as a function of *e–e* interaction and time. It is evident that the current density decreases with increasing *e–e* interaction at the smaller time scale, but after the current reaches its steady state, the effect of *e–e* interaction is very small.

### Upward Pulse modulation

**Figure 10:**
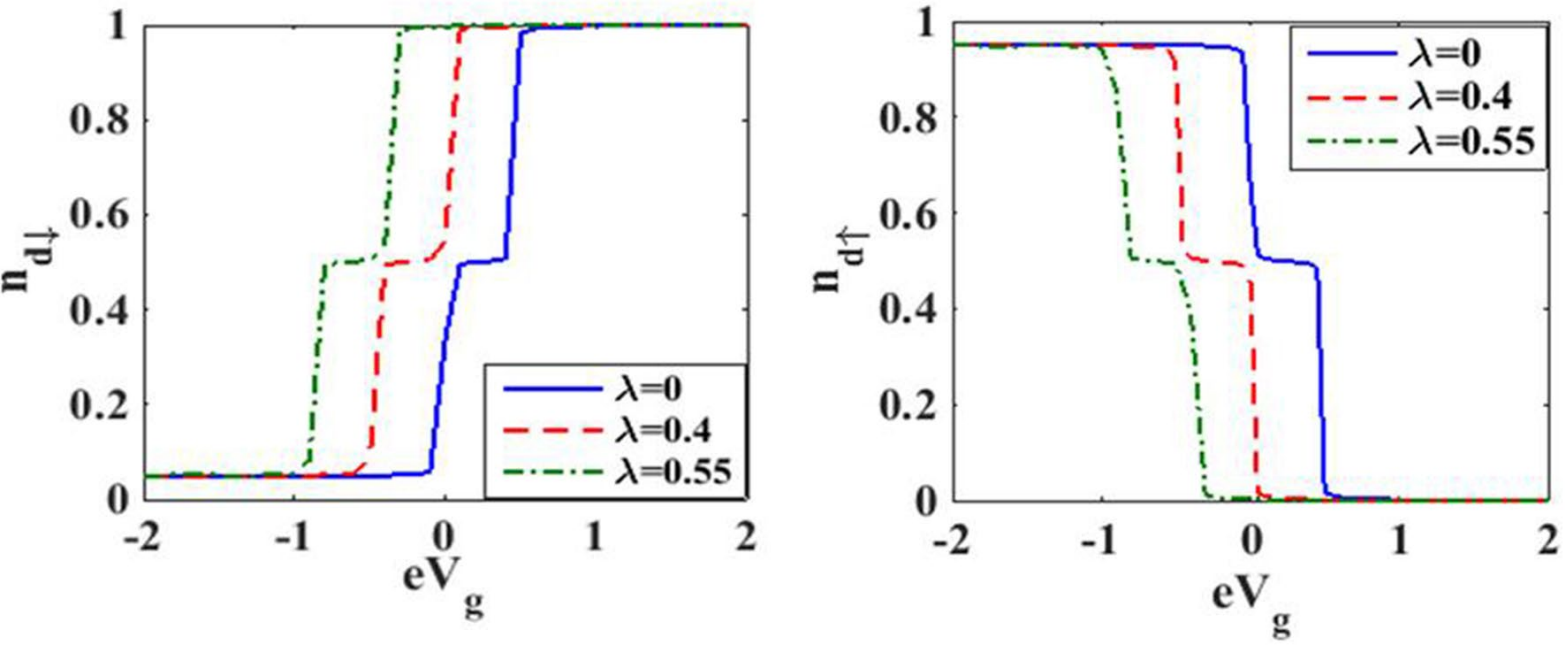
Dot occupancy versus ${eV}_{g}$ for various values of $\lambda$ at $t=10.$

We now present the results for the upward pulse modulation for which we set the values of the system parameters as:$\Gamma =0.01, e{V}_{g}=0.6, {eV}_{b}=0.5, {k}_{B}T=0, \hslash {\omega }_{0}=1$, ${\Delta }_{D}=0$ and ${\Delta }_{S}=5$. Figure 10 shows the effects of *e–p* coupling on the dot occupancy as a function of gate voltage. Again the staircase structure is visible, though the behavior of ${N}_{d\uparrow }$ and ${N}_{d\downarrow }$ are opposite as a function of tunable parameter ${eV}_{g}.$ However, the *e–p* coupling strength reduces the occupancy for both up and down spin electrons to maintain the total occupancy is equal to one.

**Figure 11:**
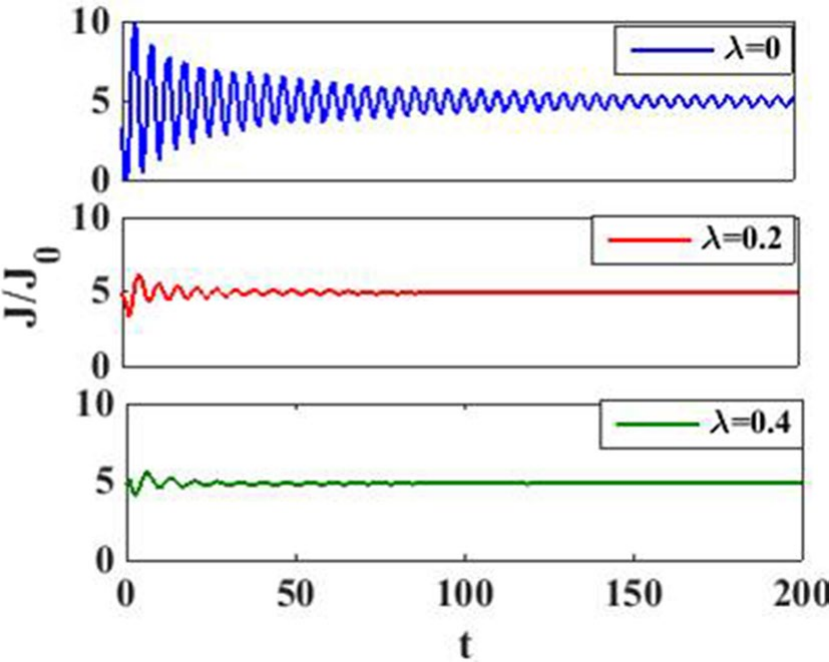
$J/J_{0}$ versus $t$ for various values of $\lambda$ at $eV_{g} = 0.6$.

We show the current density behavior as a function of time with different *e–p* coupling values in Fig. 11. The *e–p* interaction is seen to reduce the amplitude current oscillations and transient time as observed in the case of harmonic pulse modulation.

**Figure 12:**
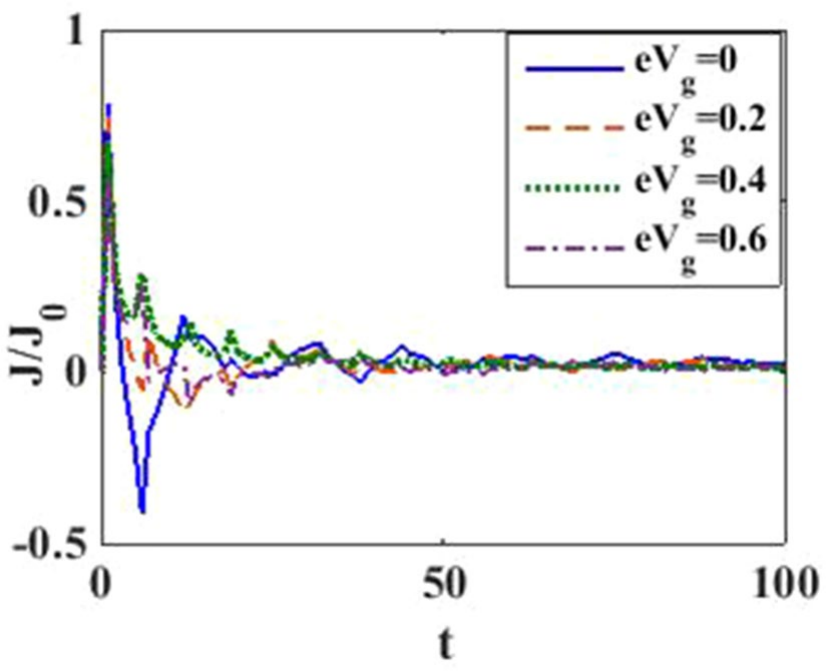
$J/J_{0}$ versus $t$ for various values of $eV_{g}$ at $\lambda = 0.6$.

**Figure 13:**
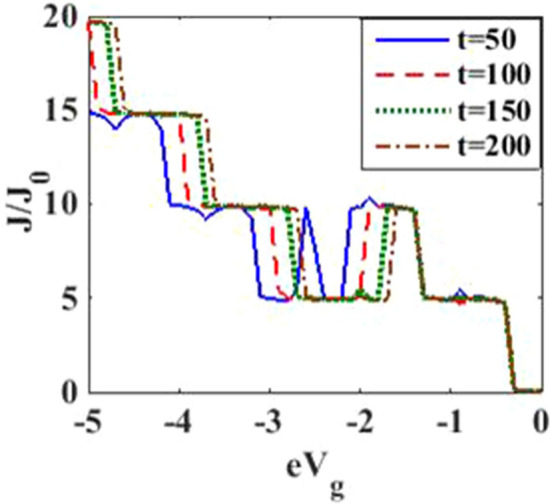
$J/J_{0}$ versus $eV_{g}$ for various values of time at $\lambda = 0.6$ and $eV_{b} = 0.5$.

**Figure 14:**
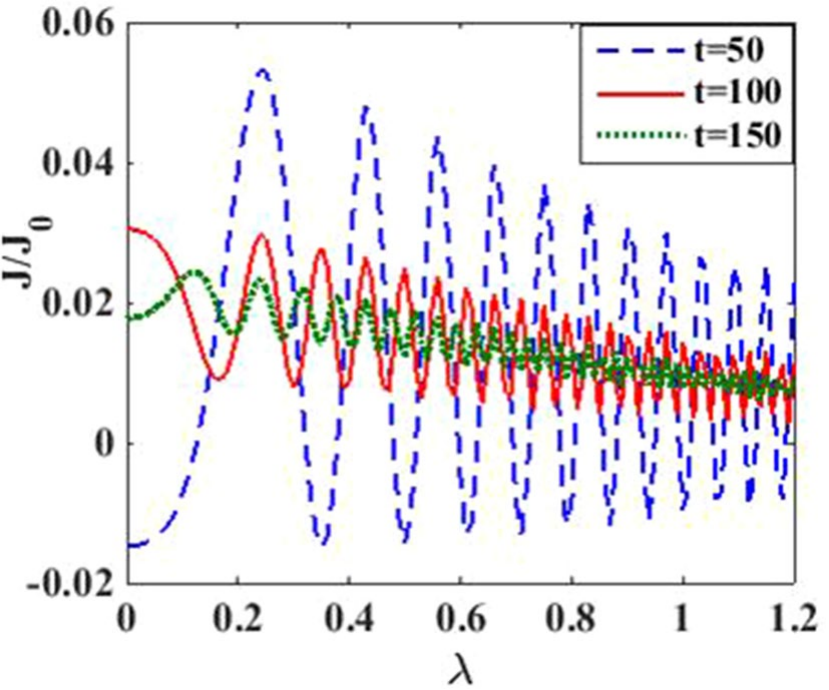
$J/J_{0}$ versus $\lambda$ for various values of $t$ at $eV_{g} = 0.3$ and $eV_{b} = 0.5$.

**Figure 15:**
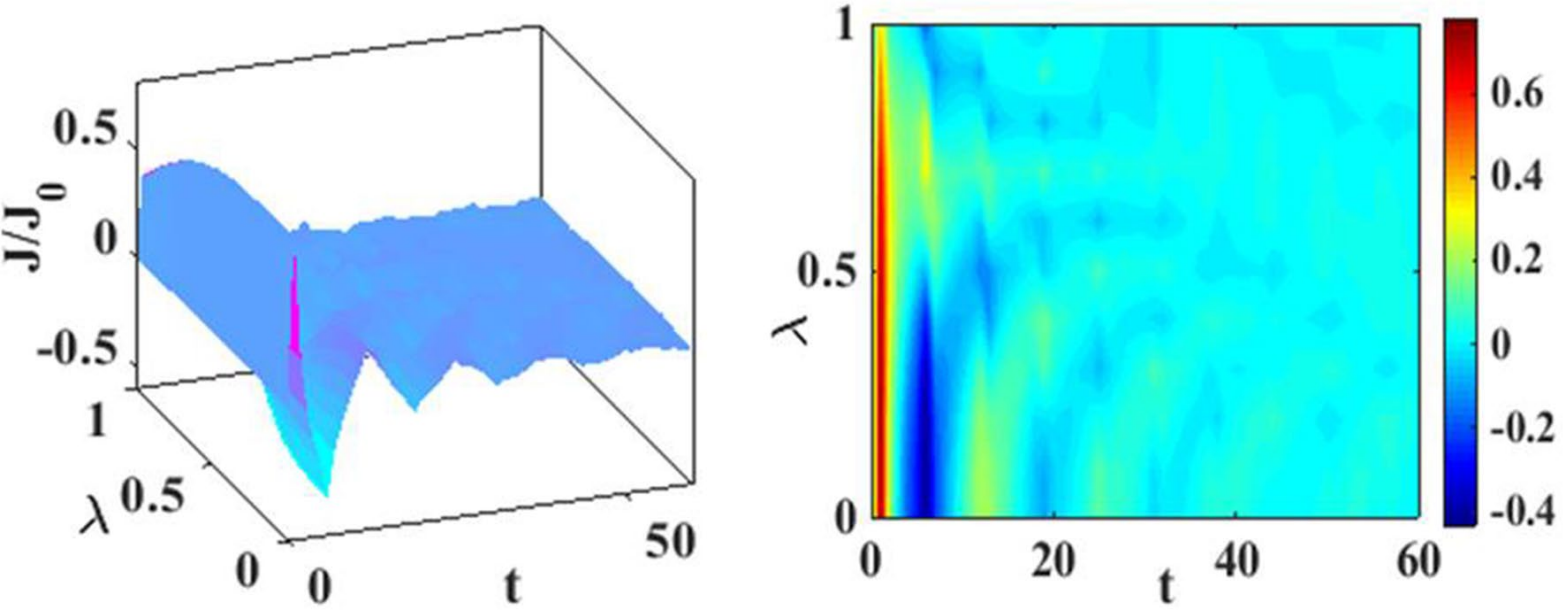
Current density as a function of $t$ and $\lambda$. Three-dimensional plot (left) and contour map (right).

**Figure 16:**
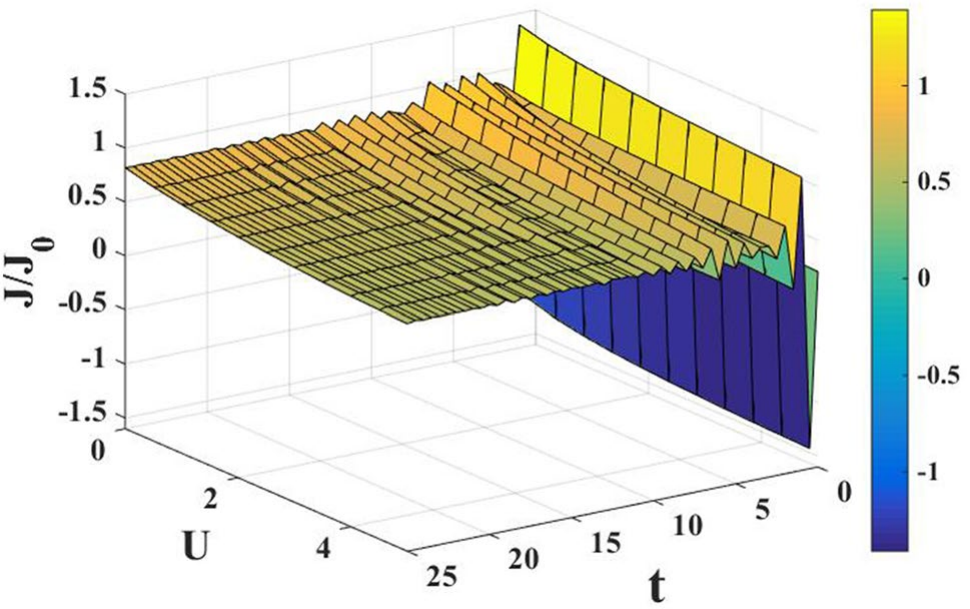
The contour map of the current density as a function of $t$ and $U$ at $\lambda = 0.4, eV_{g} = 0.6$.

In Fig. 12, we plot the current density as a function of time with different gate voltages and at a particular value of *e–p* coupling. Comparison of the transient time for several values of the gate voltage shows that it is smaller at higher values of the gate voltage. Thus the gate voltage also reduces the transient time. For a better understanding in Fig. 13, we show the current density versus gate voltage result for different $t$ values. Clearly, the $J/J_{0}$ reduces with staircase structure with increasing gate voltage because as the gate voltage increases, the dot level moves out of the bias range. Also, the width of the plateau region with constant current is increasing for higher values of time. In the plateau region, the device can be used for the switching applications. We plot the current density as a function of $\lambda$ at different times in Fig. 14. It is interesting to see that the oscillations in the $J/J_{0}$ reducing with increasing the *e–p* interaction strength and at the same time, current density decreasing with $\lambda$. Figure 15 represents the three-dimensional and contour plot of current density as a function of time and $\lambda$. Thus, one can conclude that $\lambda$ reduces the transient time and also as $\lambda$ increases, the current density saturates when $t$ is beyond 50. Thus, one can conclude that $\lambda$ reduces the transient time and current becomes almost constant at higher values of $\lambda$ due to a strong polaronic effect. In Fig. 16, we have shown the contour map of the current density as a function of *e–e* interaction and the time to see the parameter region to obtain the steady state current.

## Conclusions

In this work, we have studied the transient dynamics of an SMT device with *e–e* and *e–p* interactions and quantum dissipation for the harmonic pulse and upward pulse modulations. The system is modeled by the Anderson-Holstein-Caldeira-Legget Hamiltonian. The linear interaction of dot phonons with the substrate phonons, which introduces the dissipation, is treated with an exact transformation that essentially modifies the QD phonon frequency. Next, the *e–p* interaction is decoupled from the Hamiltonian by applying the Lang-Firsov unitary transformation and finally, we employed Keldysh formalism to calculate the current density expression. We investigated the variation of the transient time with respect to the system parameters like *e–p* interaction strength, damping coefficient and $eV_{g}$ for both the harmonic and upward pulse modulations. The most important result that we have perceived is that the transient time decreases with increasing the *e–p* and *e–e* interactions. Also, we have shown the parameter region with constant current, where the device can be used for switching applications. The current density decreases with increasing *e–p* interaction strength due to the polaronic effect. Our theoretical results can be easily tested with the experiments on a single molecular transistor, nanomolecular switching devices.
